# Evolutionary Analysis of *GH3* Genes in Six *Oryza* Species/Subspecies and Their Expression under Salinity Stress in *Oryza sativa* ssp. *japonica*

**DOI:** 10.3390/plants8020030

**Published:** 2019-01-24

**Authors:** Weilong Kong, Hua Zhong, Xiaoxiao Deng, Mayank Gautam, Ziyun Gong, Yue Zhang, Gangqing Zhao, Chang Liu, Yangsheng Li

**Affiliations:** State Key Laboratory for Hybrid Rice, College of Life Sciences, Wuhan University, Wuhan 430072, China; Weilong.Kong@whu.edu.cn (W.K.); zhonghua0103@whu.edu.cn (H.Z.); 2017102040003@whu.edu.cn (X.D.); mayankgautam@whu.edu.cn (M.G.); Gziyun@whu.edu.cn (Z.G.); Yue.Zhang-@whu.edu.cn (Y.Z.); zhaogangqing@whu.edu.cn (G.Z.); lchang@whu.edu.cn (C.L.)

**Keywords:** rice, *Oryza* species, GH3, salinity stress, RNA-seq, qRT-PCR, gene duplication

## Abstract

Glycoside Hydrolase 3 (*GH3*), a member of the Auxin-responsive gene family, is involved in plant growth, the plant developmental process, and various stress responses. The *GH3* gene family has been well-studied in *Arabidopsis thaliana* and *Zea mays*. However, the evolution of the *GH3* gene family in *Oryza* species remains unknown and the function of the *GH3* gene family in *Oryza sativa* is not well-documented. Here, a systematic analysis was performed in six *Oryza* species/subspecies, including four wild rice species and two cultivated rice subspecies. A total of 13, 13, 13, 13, 12, and 12 members were identified in *O. sativa* ssp. *japonica*, *O. sativa* ssp. *indica*, *Oryza rufipogon*, *Oryza nivara*, *Oryza punctata*, and *Oryza glumaepatula*, respectively. Gene duplication events, structural features, conserved motifs, a phylogenetic analysis, chromosome locations, and Ka/Ks ratios of this important family were found to be strictly conservative across these six *Oryza* species/subspecies, suggesting that the expansion of the *GH3* gene family in *Oryza* species might be attributed to duplication events, and this expansion could occur in the common ancestor of *Oryza* species, even in common ancestor of rice tribe (*Oryzeae*) (23.07~31.01 Mya). The RNA-seq results of different tissues displayed that *OsGH3* genes had significantly different expression profiles. Remarkably, the qRT-PCR result after NaCl treatment indicated that the majority of *OsGH3* genes play important roles in salinity stress, especially *OsGH3-2* and *OsGH3-8*. This study provides important insights into the evolution of the *GH3* gene family in *Oryza* species and will assist with further investigation of *OsGH3* genes’ functions under salinity stress.

## 1. Introduction

Auxin is crucial for various aspects of plant growth and development, including signaling transport, plant metabolism, apical dominance, and shoot elongation [1,2]. Auxin’s production, storage, degradation, and migration to the region of its action are tightly regulated both spatially and temporally [2,3]. Auxin-regulated genes include different Auxin-responsive families, such as Auxin/Indole-3-Acetic Acid genes (*AUX/IAA*s act as repressors), Auxin Response Factor genes (*ARF*s act as transcription activators), Small Auxin Up RNA genes (*SAUR*s regulate the auxin-signaling pathway), and Gretchen Hagen 3 genes (*GH3*s) [3,4]. The GH3 enzyme family conjugates amino acids to chemically diverse compounds, such as Jasmonic acid (JA), Indole-3-acetic acid (IAA), and Salicylic acid (SA) at the cellular level and modulates crosstalk among JA, IAA, and SA, which in turn is involved in plant physiological processes [1,5,6,7,8,9].

Since the first *GH3* gene was cloned from soybean [4], genome-wide analyses have identified 19 *GH3* genes in *Arabidopsis thaliana*, 13 in *Oryza sativa* ssp. *japonica*, 13 in *Zea mays*, 15 in *Solanum lycopersicum*, 2 in *Physcomitrella patens*, and 18 in *Selaginella moellendorffii* [6,8,9,10,11,12,13]. To date, *GH3* genes have been categorized into three groups (I–III) based on sequence similarity and substrate specificities [5,9]. In *Arabidopsis*, GH3 proteins from group I, with JA and/or SA-amido synthetase activity, use JA or SA as a substrate. GH3 proteins from group II, with IAA-amido synthetase activity, have Auxin-inducible expression profiles [5,9]. GH3 proteins from group III remain largely unknown to date. *AtGH3-12/PBS3*, an acyladenylase family member, is the best-studied class III protein. It regulates SA-dependent defense response by encoding a putative serine-threonine kinase that is involved in the conjugation of glutamic acid to 4-aminobenzoate and 4-hydroxybenzoate [9]. In *O. sativa* ssp. *japonica*, the *GH3* gene family includes 13 members: four members in group I (*OsGH3-3*, *-5*, *-6*, and *-12*) and nine members in group II (*OsGH3-1*, *-2*, *-4*, *-7*, *-8*, *-9*, *-10*, *-11*, and *-13*). Of all *OsGH3* genes, *OsGH3-1*, *-2*, *-8*, and *-13* regulate the modulation of crosstalk between the IAA [13,14], JA, and SA signaling pathways for rice tolerance to biotic or/and abiotic stresses [13,15,16,17]. *OsGH3-2* modulates Auxin and Abscisic acid (ABA) levels, triggering drought and cold tolerances [18]. Rice overexpressing *OsGH3-2* has several filamentous roots and a lower number of lateral roots, indicating that *OsGH3-2* is involved in the regulation of lateral root development [13,18]. The ‘Auxin-*miR167*-*ARF8*-*OsGH3-2’* signaling pathway was proposed by Yang et al. (2009). It assumes that *OsGH3-2* is positively regulated by ARF8 (an Auxin response factor) and is negatively degraded by *miR167*. *miR167* is regulated by Auxin [19]. Previous research has also reported that *OsGH3-8* can be induced by Auxin, SA, and JA, and plays an important role in rice growth and disease resistance [15]. *OsGH3-8* is a common downstream target of *OsMADS1* and *OsMADS6* and controls rice floret fertility [20,21]. Recently, Dai et al. (2018) reported that *OsGH3-8* can modulate the plant architecture by the *miR156f*-*OsSPL7*-*OsGH3-8* pathway in rice [22]. Additionally, *OsGH3-13* improves rice drought tolerance by downregulating IAA content [17].

Cultivated rice (*O*. *sativa* L.) is the second-most important staple food crop worldwide [23,24,25]. *Oryza* species, with great economic value, can provide major genes for the hybrid rice revolution and sustainable rice production [26]. However, the characterization and evolution relation of *GH3* genes in *Oryza* species are still largely unknown. The function of the *GH3* gene family in *Oryza sativa* under salinity stress remains unclear. In this study, we performed a genome-wide analysis of *GH3* gene family members to analyze gene structure, protein motifs, chromosomal localizations, and collinear gene pairs among six *Oryza* species/subspecies. Furthermore, functional annotations and a cis-acting element analysis of *OsGH3* genes were performed. Finally, the expression patterns of 13 *OsGH3* genes in different tissues and under salinity stress were examined using RNA-seq and qRT-PCR. The present study may provide a better understanding of the evolution of *GH3* genes in *Oryza* species and functions of *OsGH3* genes in *O. sativa* under salinity stress.

## 2. Materials and Methods

### 2.1. Plant Materials

Rice ‘Nipponbare’ (*O. sativa* ssp. *japonica*) was chosen for the quantitative real-time RT-PCR (qRT-PCR). After 2 days of germination in water at 37 °C, seeds were grown in containers with sponges as supporting materials in Yoshida solution with 60% relative humidity and with a light and temperature regime of 14 h/10 h, light/dark, 30 °C/22 °C. Three-leaf stage seedlings were transferred to 200 mM NaCl Yoshida solution for salt treatment. Then, leaves and roots of treatment/control seedlings were collected at 0, 3, 6, 12, and 24 h for RNA extraction. For each biological replicate, 15 seedlings were collected and mixed to minimize the effect of transcriptome unevenness among rice seedlings. Total RNA was extracted using the TRIzol method and reverse transcribed into cDNA using the PrimeScript RT reagent Kit (TakaRa, Dalian, China).

### 2.2. Identification of the GH3 Genes

Potential members of the *GH3* gene family were identified based on the Hidden Markov model (HMM) and BLAST homology searches [27,28]. Protein and nucleotide sequences of wild rice (*Oryza rufipogon*, version: OR_W1943.39; *Oryza nivara*, version: v1.0; *Oryza punctata*, version: v1.2; *Oryza glumaepatula*, version: v1.5) and cultivated rice (*O. sativa* ssp. *indica*, version: ASM465v1, version: R498) were downloaded from EnsemblPlants (http://plants.ensembl.org/index.html) and MBKBASE (http://www.mbkbase.org/R498/). GH3 protein sequences of *Arabidopsis* and rice (*O. sativa* ssp. *japonica*) were downloaded from the TAIR Database (https://www.arabidopsis.org/) and RiceData (http://www.ricedata.cn/gene/) as query sequences [11,12,29]. These query sequences were used to search for GH3 protein sequences in five *Oryza* species’ protein databases using local ncbi-blast-2.7.1+ (ftp://ftp.ncbi.nlm.nih.gov/blast/executables/blast+/LATEST) in the Blastp method with a cut-off E-value of e^−5^. Then, PF03321 was downloaded from Pfam (http://pfam.xfam.org/) and PF03321 was used to query the *Oryza* species’ proteins database using HMMER 3.0 software (http://hmmer.org/) [27]. Since the GH3 domain is longer than 400, protein sequences with a length of less than 400 were deleted in this study. Finally, the GH3 domains of all the nonredundant protein sequences were verified by SMART (http://smart.embl-heidelberg.de/) and Pfam (http://pfam.xfam.org/search/sequence) [8,27].

### 2.3. Phylogenetic Analysis

Multiple sequences alignments with *Arabidopsis* and *O. sativa* ssp. *japonica* were conducted by Clustal W, separately. A phylogenetic tree was generated by MEGA 6.0 using the Neighbor Joining (NJ) method with 1000 bootstrap replicates [27,28,30,31]. Subsequently, *GH3* sequences of five *Oryza* species were systematically named based on the clustering results and names from previous studies [11,12,31].

### 2.4. Analysis of Gene Structure and Conserved Motifs

The exon/intron structure of *GH3* genes was analyzed by comparing the coding DNA sequences (CDS) and the genomic sequences using the GSDS 2.0 (http://gsds.cbi.pku.edu.cn/). The Multiple Expectation Maximization for motif Elicitation (MEME, http://meme-suite.org/tools/meme) tool was used to predict conserved motifs of GH3 proteins with these parameters: the number of motifs (20) and other parameters (default values) [27,29,30]. Gene structure and conserved motifs were visualized using TBtools [32].

### 2.5. Analysis of Chromosome Locations, Gene Duplication Events, and Ka/Ks Values

Chromosome locations of *GH3* genes were obtained from GFF3 files. Gene duplication patterns of *GH3* genes were analyzed by the ‘duplicate_gene_classifier’ script in MCScanX (http://chibba.pgml.uga.edu/mcscan2/) with the default parameters [28,33]. Chromosome locations and gene duplication events were visualized using Circos software (http://circos.ca/) [34]. The synonymous (Ks) and nonsynonymous (Ka) substitution rates were estimated using DnaSP 5.0 (http://www.ub.edu/dnasp/) [35]. Divergence time (T) was estimated by T = Ks/(2 × 9.1 × 10^−9^) × 10^−6^ million years ago (Mya) [28,36].

### 2.6. Microsynteny Analysis, Cis-Acting Element Analysis, and Functional Annotation Analysis

The microsynteny between six *Oryza* species/subspecies was analyzed by MCScanX with the default parameters. Collinear gene pairs between six *Oryza* species/subspecies were drawn using Circos software [34]. Cis-acting regulatory elements (Cis-elements) of each promoter (2 Kbp upstream from the translation start site, ATG) of the *OsGH3* gene were analyzed by the PLANTCARE program (http://bioinformatics.psb.ugent.be/webtools/plantcare/html) [37].The functional annotations of the OsGH3 proteins (OsGH3s) were performed using Blast2GO software [38]. The KEGG pathways of OsGH3s were carried out using the KEGG database (http://www.kegg.jp) [39].

### 2.7. Expression Analysis and Co-Expression Network Analysis of OsGH3 Genes Based on the RNA-seq Datasets from Different Tissues

Raw datasets (SRX100741, SRX100757, SRX100743, SRX100745, SRX100746, SRX100747, SRX100749, SRX100753, SRX100754, SRX100756, SRX100755, SRR042529, and SRX016110) were obtained from the NCBI (https://www.ncbi.nlm.nih.gov/). These datasets were used to analyze the expression profiles of *OsGH3* genes in different tissues (leaves-20 days, post-emergence inflorescence, pre-emergence inflorescence, anther, pistil, seed-5 days after pollination (DAP), embryo-25 DAP, endosperm-25 DAP, seed-10 DAP, shoots, and seedling four-leaf stage). Tophat2 was applied to map raw data to the reference genome (MSU 7.0, http://rice.plantbiology.msu.edu/). Then, Cufflinks software was adopted to calculate gene expression [40]. The R package was used to generate quantitative differences in the expression level of each gene based on the log2^FPKM^ values [29].

A co-expression network in different tissues was constructed based on RNA-seq datasets using the Comparative Co-Expression Network Construction and Visualization tool (CoExpNetViz, http://bioinformatics.psb.ugent.be/webtools/coexpr/) with previously reported parameters [27]. The Co-expression network was visualized using Cytoscape V.3.1.0. The correlation coefficient >0.50 or < −0.50 was limited.

### 2.8. Expression Analysis of OsGH3 Genes under Salinity Stress by qRT-PCR

Primers of *OsGH3* genes were designed by Primer 5.0 in specific regions or 3’–UTR regions (Primers in Table S1) [29]. The qRT-PCR reaction (10 μL) was formulated using ChamQ™ SYBR^®^ Color qPCR Master Mix (Vazyme, Shanghai, China). qRT-PCR was carried out in 96-well plates on a CFX96 Touch™ Real-Time PCR Detection System (Bio-Rad, Hercules, CA, USA). *Ubi* (*LOC_Os03g13170*, encodes the ubiquitin fusion protein) was used as an internal control. The average threshold cycle (Ct) from three biological replicates was used to determine the fold change of *OsGH3* gene expression by the 2^−ΔΔCT^ method [29].

## 3. Results

### 3.1. Identification and Classification of the GH3 Gene Family

**Figure 1:**
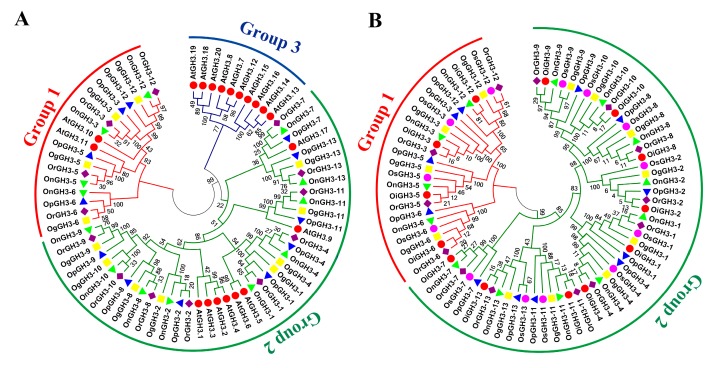
(**A**) A phylogenetic tree of GH3 protein sequences from five *Oryza* species and *Arabidopsis*. (**B**) A phylogenetic tree of GH3 protein sequences from six *Oryza* species/subspecies. Clustal W is used for multiple sequence alignment. MEGA 6.0 is adopted for phylogenetic reconstruction by using the Neighbor Joining (NJ) clustering method. Bootstrap numbers (1000 replicates) are shown. Different colors of circles represent different subfamilies. The different species are indicated by different shaped markers.

A total of 13, 13, 13, 12, and 12 members were identified in *O. sativa* ssp. *indica*, *O. rufipogon*, *O. nivara*, *O. punctata*, and *O. glumaepatula*, respectively. Based on the homologous sequence cluster result with *Arabidopsis* and *O. sativa* ssp. *japonica*, all *GH3* genes were grouped into two groups (Table 1 and Figure 1): group1 and group2. In *O. sativa* ssp. *indica*, *O. rufipogon*, and *O. nivara*, four members of *GH3s* belonged to group1, and nine members of *GH3s* belonged to group2. In *O. punctata* and *O. glumaepatula*, four members of *GH3s* belonged to group1, and eight members of *GH3s* belonged to group2 (Table 1 and Figure 1). We found that the classification, chromosome locations, and number of *GH3* genes were strictly conservative among these six *Oryza* species, and small differences in the number of GH3 genes could be produced from assembly or sequencing errors because these two incomplete sequences were also identified, namely *OpGH10* (ID: OPUNC07G18660.1) in *O. punctata* and *OgGH7* (ID: OGLUM06G14160.1) in *O. glumaepatula*. Considering this reason, these two incomplete sequences were removed in further research. Interestingly, no members of group III were found in five *Oryza* species, while 10 members of group III were found in *Arabidopsis* (Figure 1).

### 3.2. Gene Structure and Conserved Motif Analysis

**Figure 2:**
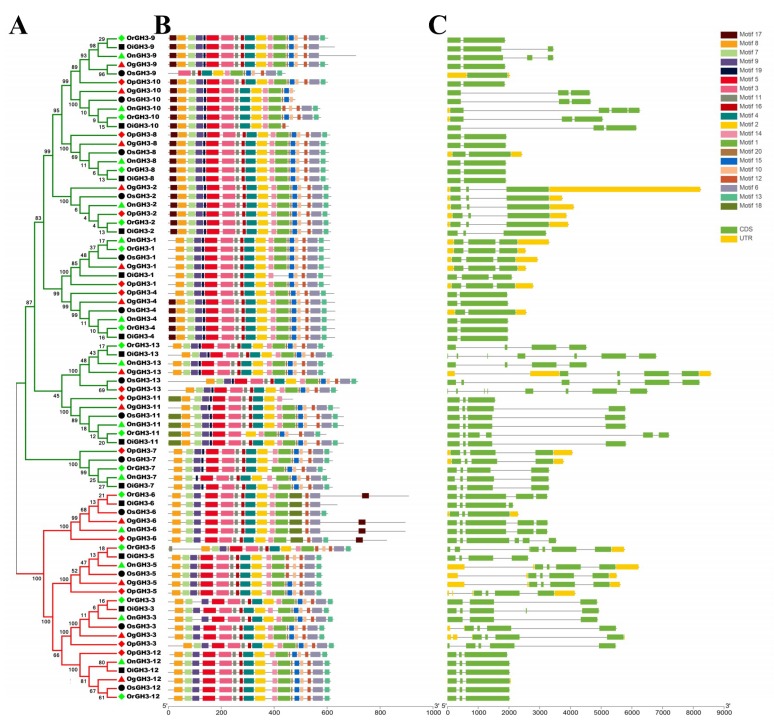
The phylogenetic tree (**A**), motif compositions (**B**), and exon/intron structure (**C**) of the *GH3* genes in six *Oryza* species/subspecies. (**A**) Sequence alignments and the NJ-Phylogenetic trees were made using ClustalW and MEGA 6.0, respectively. A bootstrap number (1000 replicates) is adopted. The red and green colors in the phylogenetic tree represent group1 and group2, respectively. (**B**,**C**) The widths of the grey bars represent the relative lengths of genes and proteins. The green boxes and grey lines display exons and introns, respectively.

Earlier studies have shown that gene structure diversity can provide the primary power for the evolution of multigene families [7,27,28,29,36]. Thus, an exon/intron analysis was performed to obtain more insights into the structural diversity of *GH3*s in *Oryza* species (Figure 2). The analysis results showed that the intron number of *GH3*s in six *Oryza* species/subspecies ranged from 1 to 7, *GH3-4* contained the fewest introns (1), and *GH3-13* contained the most introns (3–7) (Figure 2). Furthermore, conserved motifs of 63 GH3 proteins were analyzed by MEME. As a result, 20 conserved motifs were identified, and the 63 GH3 proteins showed a similar conserved motifs arrangement. Notably, we found that *GH3*s from the same group showed differences in the number and the length of exons/introns. These results suggest that the gene function from the similar group has diversified. In short, the gene structure and conserved motif analysis of *GH3*s strongly supports the reliability of the group classification of *GH3s* in Figure 1.

### 3.3. Chromosome Locations, Duplication Events, Selection Pressure, and Microsynteny Analysis

**Figure 3:**
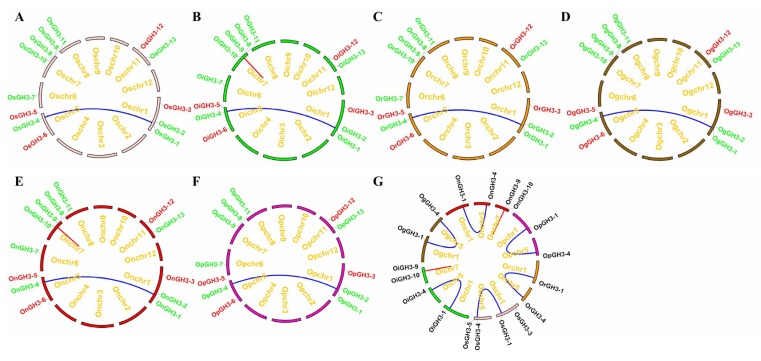
The chromosome location and duplication events of *GH3* genes in six species/subspecies (**A–F**), and duplication events in six *Oryza* species/subspecies (**G**). Os represents *Oryza sativa* ssp. *japonica*. Oi represents *Oryza sativa* ssp. *indica*. Or represents *Oryza rufipogon*. On represents *Oryza nivara*. Op represents *Oryza punctata*. Og represents *Oryza glumaepatula*. The chromosomes of different *Oryza* species/subspecies are shown by different colors. The location of each *GH3* gene is marked with a grey line using Circos software. The whole genome duplication (WGD) or segmental duplication/Tandem duplication gene pairs are linked by blue/red lines. The red and green genes in A–F belong to group1 and group2, respectively.

**Figure 4:**
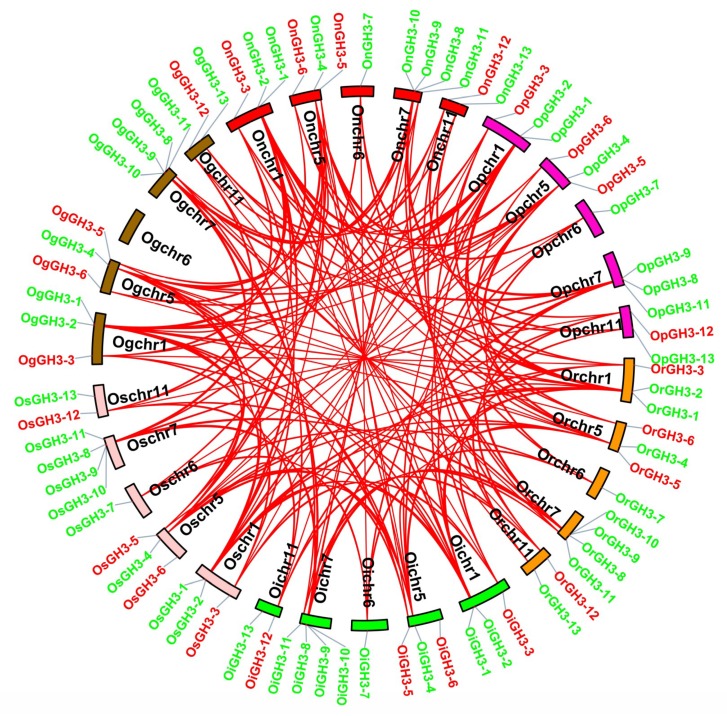
The collinear gene pairs across six *Oryza* species/subspecies. The chromosome colors and abbreviations of species are the same as in Figure 3. The red lines represent collinear gene pairs.

The chromosome locations result showed that 13 *GH3* genes were unevenly mapped on 12 chromosomes among the six species and subspecies. Four *GH3* genes (30.77%) mapped on chromosome (Chr) 7, three *GH3* genes (23.08%) mapped on Chr1 and Chr5, two *GH3* genes (15.38%) mapped on Chr11, and one *GH3* gene (7.69%) mapped on Chr6. No *GH3* gene was found on Chr2, Chr3, Chr4, Chr8, Chr10, and Chr12 (Figure 3A–F). These uneven distribution patterns of the *GH3* gene family have also been observed in *Arabidopsis*, maize, tomato, and potato [5,6,8,9,10]. Moreover, we discovered six pairs of segmental duplication events and two pairs of tandem duplication events in the six *Oryza* species/subspecies (Figure 3). Interestingly, *GH3-1* and *GH3-4* segmental duplication events were found in every species/subspecies (Figure 3). However, *GH3-9* and *GH3-10* tandem duplication events were only found in *O. sativa* ssp. *indica* and *O. nivara* (Figure 3B,E,G). Our findings suggest that duplication events were major factors determining the expansion of the *GH3* gene family. Next, Ka/Ks values of duplicated *GH3* gene pairs were calculated to evaluate the driving force underlying the *GH3* gene’s evolution. Ka/Ks >1, <1, and =1 mean a positive selection, a negative selection, and a neutral selection, respectively. The Ka/Ks results showed that the Ka/Ks values of eight duplicated *GH3* genes ranged from 0.1245 to 0.2070 and were less than 1 (Table 2). These results demonstrated that the duplicated *GH3* genes were under a strong negative selection during the evolution process. The segmental duplication events of these six gene pairs were estimated to occur between 23.20 and 31.01 Mya (Table 2). Besides this, to further understand the evolutionary process of the *GH3* genes in *Oryza* species, a microsynteny analysis was conducted among the six *Oryza* species/subspecies. In total, 169 collinear gene pairs were identified (Figure 4, Table S2).

### 3.4. Analysis of Cis-Elements in OsGH3 Genes

**Figure 5:**
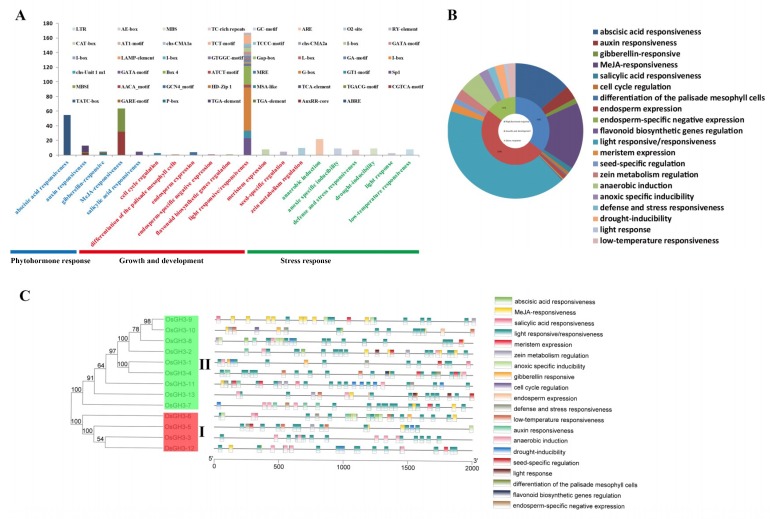
Identification of cis-acting elements in all *GH3* genes of *Oryza sativa* ssp. *japonica*. (**A**) The different bars represent different primary categories, the different characters represent different secondary categories, and the different colors in histograms represent the number of different promoter elements in each secondary category. (**B**) Pie charts of different sizes indicate the ratio of each primary/secondary category. (**C**) The different groups of *GH3* genes in the phylogenetic tree are shown by different colors. The differently colored boxes represent the different secondary categories.

The Cis-elements present in the stress-responsive gene promoters can provide an important insight into the stress response of plants [28,41,42]. Thus, the PlantCare database was used to identify the Cis-elements present in the promoter regions in the *OsGH3* genes. Our results showed a high frequency of occurrence of Cis-elements in *OsGH3* genes (Figure 5) and that 47 Cis-elements were identified. These Cis-elements can be divided into 3 primary categories, including 20 secondary categories based on the previously described functional categories [43]. The cis-elements in the growth and development primary category showed a higher frequency of occurrence than that in the stress response and phytohormone response primary category (Figure 5A,B). In the growth and development primary category, the number of light responsive/responsiveness secondary category elements exceeded 160 and contained 23 types of elements (Figure 5A). In the phytohormone response primary category, the MeJA-responsiveness secondary category was the top secondary category (64), including the CGTCA-motif and the TGACG-motif, followed by the abscisic acid responsiveness secondary category (55), including the ABRE element (Figure 5A). In the phytohormone response primary category, the top three secondary categories were the anaerobic induction, anoxic specific inducibility, and drought inducibility secondary categories, respectively (Figure 5A). The result of the Cis-element position analysis showed that Cis-elements are unevenly distributed on all genes, and several Cis-elements were preferentially present on individual genes; for instance, the *OsGH3-9* promoter regions had a lot of MeJA-responsive Cis-elements (Figure 5C). Thus, we proposed that *OsGH3* genes have the potential to improve stress tolerances because the *OsGH3* genes contain several biotic/abiotic stress motifs in their promoter regions. It could also be further speculated that *OsGH3-9* plays an important role in MeJA-related processes.

### 3.5. Functional Annotations Analysis of the OsGH3 Proteins

**Figure 6:**
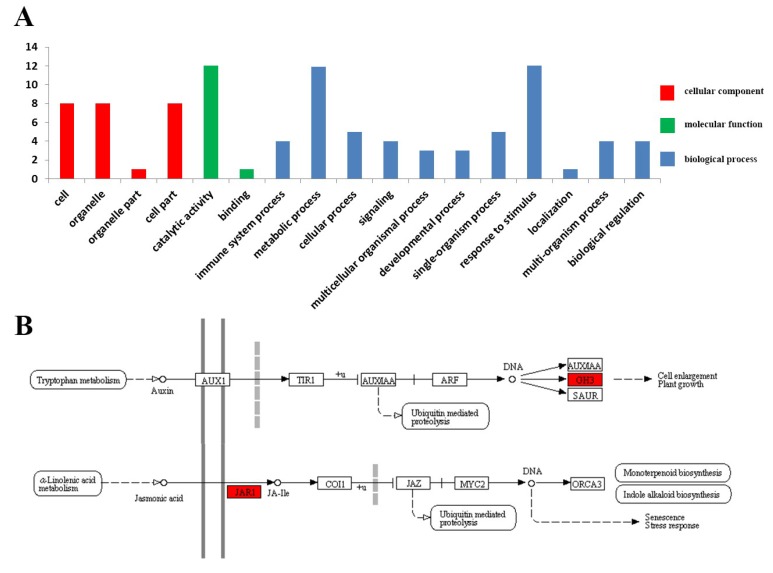
Gene ontology classification and KEGG pathway annotation of *OsGH3* genes. (**A**) The Y-axis indicates the actual gene number and the X-axis indicates three categories (cellular component, molecular function, and biological process). (**B**) The *OsGH3*-gene-related KEGG pathways are marked in red.

The results of the GO annotation and KEGG pathway analysis provide us with a better understanding of the biological functions of the different OsGH3 proteins. In this study, the GO annotation result revealed that 13 OsGH3 proteins were divided into 17 specific classes represented under the functional domains molecular functions, cellular components, and biological processes. Catalytic activity, metabolic process, and response to stimulus were predominant among the above-specified classes (Figure 6). KEGG pathway results revealed that nine OsGH3 proteins (eight OsGH3 proteins belonged to group2, except for OsGH3-6) were enriched on the Auxin pathway, while three OsGH3 proteins (belonging to group 1) were enriched on the JA pathway. These results support previous reports that GH3 proteins from group I, with JA and/or SA-amido synthetase activity, use JA or SA as a substrate. GH3 proteins from group II, with IAA-amido synthetase activity, have Auxin-inducible expression profiles [5,9].

### 3.6. Expression Analysis of OsGH3 Genes in Different Tissues and under Salinity Stress

**Figure 7:**
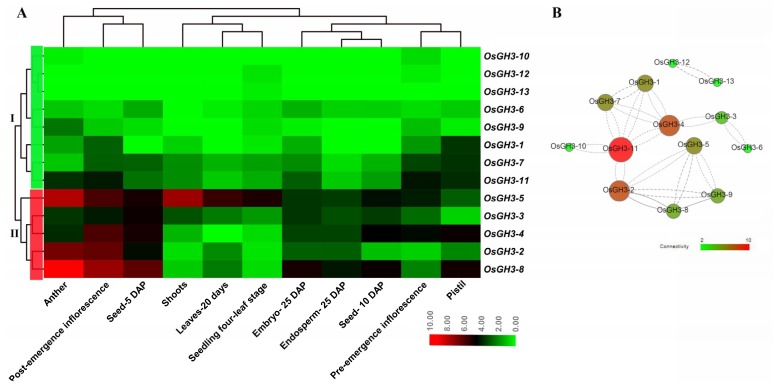
Expression profiles of *OsGH3* genes in different tissues (**A**) and a co-expression network diagram of *OsGH3* genes in different tissues. (**A**) The color scale at the bottom of the image represents log_2_^FPKM^; red indicates a high level; and green represents a low level of transcript abundance. (**B**) The Correlation from weak to strong is shown by dotted line to solid line. Connectivity from weak to strong is shown from green to red. DAP, days after pollination.

The expression analysis results revealed that *OsGH3* genes showed different expression patterns in different tissues (Figure 7, Table S3). All *OsGH3* genes clustered into two major groups based on expression levels (Figure 7A). The genes in Group I (including eight members) showed low expression levels in all tissues, whereas the genes in Group II (including five members) had relatively high expression levels in some tissues. For example, *OsGH3-5* showed a high expression level in anther and shoots. *OsGH3-4* displayed relatively high expression levels in seed-5 DAP. *OsGH3-2* exhibited a high expression level in anther and post-emergence inflorescence. *OsGH3-8* exhibited a high expression level in anther, post-emergence inflorescence, and seed-5 DAP (Figure 7A). Besides this, the co-expression network results indicated that there was a strong co-expression relationship network within the *GH3* family’s genes and that *OsGH3-11*, *OsGH3-2*, and *OsGH3-4* were Hub genes in this network.

**Figure 8:**
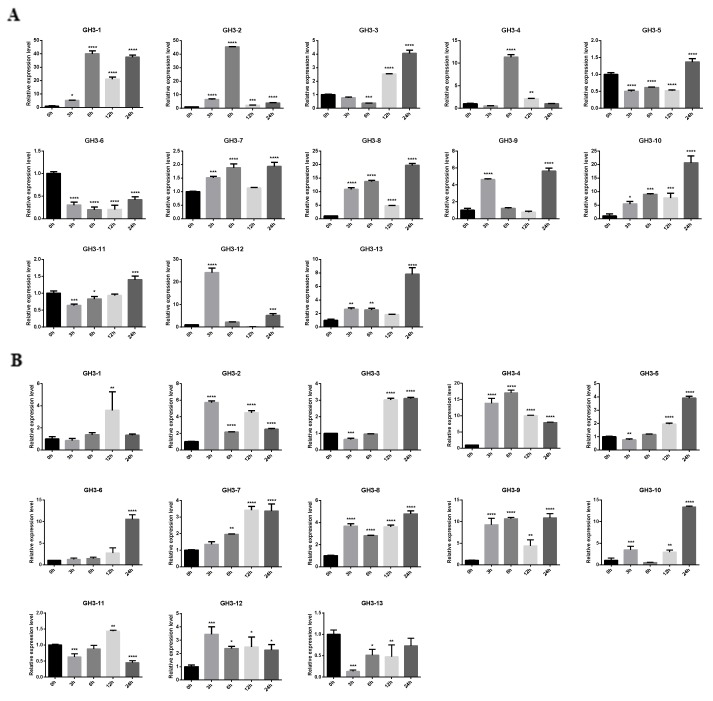
The expression pattern of the 13 *OsGH3* genes in ‘Nipponbare’ seedling leaf (**A**) and root (**B**) after NaCl treatment for 0 h, 3 h, 6 h, 12 h, and 24 h. The 2^−ΔΔCT^ method was adopted to calculate the fold change of *OsGH3* gene expression from three biological replicates. The error bars show the standard deviations of the three independent qRT-PCR biological replicates. * represents a significant difference relative to the 0 h group (*p* < 0.05).

It is common knowledge that salinity stress is a serious threat to crop yield worldwide [44]. The expression pattern under salinity stress can provide crucial clues to help us identify *OsGH3* genes’ functions. Hence, a qRT-PCR analysis at different time points after NaCl treatment was carried out. After NaCl treatment, the expression levels of all *OsGH3* genes showed significant changes. The expression patterns of all *OsGH3* genes in roots were different from that in leaves (Figure 8). In leaves, the expression levels of *OsGH3-1*, *OsGH3-2*, *OsGH3-8*, and *OsGH3-10* were upregulated at all of the tested points and reached the highest at 6 h, 6 h, 24 h, and 24 h, respectively, whereas *OsGH3-3*, *OsGH3-4*, *OsGH3-7*, *OsGH3-9*, *OsGH3-12*, and *OsGH3-13* were upregulated at two or three time points (Figure 8A). In contrast, the expression levels of *OsGH3-5* and *OsGH3-11* were downregulated at two or three time points in leaves (Figure 8A). The expression level of *OsGH3-6* showed downregulation at all of the tested points in leaves (Figure 8A). In roots, the expression levels of five genes, namely *OsGH3-2*, *OsGH3-4*, *OsGH3-8*, *OsGH3-9*, and *OsGH3-12*, were upregulated at all of the tested points and reached the highest at different points, while *OsGH3-3*, *OsGH3-5*, *OsGH3-7*, and *OsGH3-10* were upregulated at two or three time points (Figure 8B). Interestingly, *OsGH3-1* and *OsGH3-13* were upregulated at only one time point, respectively 12 h and 24 h, in roots (Figure 8B). Yet, the expression levels of *OsGH3-11* and *OsGH3-13* showed downregulation at two or three time points (Figure 8B) in roots. Specifically, we observed that *OsGH3-2* and *OsGH3-8* were upregulated at all tested points, namely 3 h, 6 h, 12 h, and 24 h in leaves and 3 h, 6 h, 12 h, and 24 h in roots, while *OsGH3-6* was downregulated at 3 h, 6 h, 12 h, and 24 h in leaves and at 3 h, 6 h, and 12 h in roots (Figure 8). Overall, the expression profiles in leaves and roots of these genes were different, indicating that *OsGH3* genes have different roles under salinity stress.

## 4. Discussion

The *GH3* gene family has been identified in several plants, such as *Arabidopsis* [9], *O. sativa* ssp. *japonica* [11,12,13], *Z. mays* [7], *S. lycopersicum* [6], *P. patens* [8], and *S. moellendorffii* [8], and it has important effects on plant growth, the plant developmental process, and various stress responses. Unfortunately, the evolutionary analysis of the *GH3* gene family in *Oryza* species has not been well-studied to date. In recent years, the constantly released genomes of various wild rice species provide us with an opportunity to conduct a comprehensive analysis of this important gene family, including their gene structure, conserved motifs, a phylogenetic analysis, chromosome locations, gene duplication events, Ka/Ks ratios, and expression patterns.

The present study demonstrated that the number (13 members) and the gene structure of *GH3* genes are strictly conservative across six *Oryza* species/species. The *Oryza* species has more *GH3* genes than *Marchantia polymorpha* L. (two, mosses) [45], *Physcomitrella patens* (two, mosses) [46], *Picea abies* (seven, gymnosperms), *Amborella trichopoda* (six, angiosperms), *Prunus persica* (seven, eudicots), *Capsicum annuum* (11, eudicots), *Vitis vinifera* (nine, eudicots), *Hordeum vulgare* (five, monocots), and *Phalaenopsis equestris* (six, monocots) [8]. The number of *GH3* genes in *Oryza* species is lower than that in *Selaginella moellendorffii* (18, ferns), *A. thaliana* (17, eudicots), *Brassica rapa* (38, eudicots), *S. lycopersicum* (17, eudicots), *Glycine max* (24, eudicots), *Elaeis guineensis* (16, monocots), and *Musa acuminata* (18, monocots) [8]. These results revealed that the *GH3* genes have been expanded to different degrees in various plants. Additionally, there was no positive correlation between the number of *GH3* genes and the size of the specie genome. For instance, the genome size of *A. trichopoda* is nearly twice that of *O. sativa* ssp. *japonica*, while *O. sativa* ssp. *japonica* (13) has a larger number of *GH3* genes as compared with *A. trichopoda* (6). Therefore, we speculated that the difference in the number of *OsGH3* genes is not related to the size of the genome. Besides this, we also observed an interesting phenomenon in which group III did not exist in mosses, ferns, gymnosperms, and angiosperms, whereas group III existed in eudicots and a few monocots [8], such as *Brassicaceae* plants. Considering these results, it can be deduced that group III might be the youngest group and originated from group I or group II and that group III could be related to the adaptation of plants to specific environments because group III has a species-specific expansion in various plants.

In the six *Oryza* species/subspecies, the same duplication events (*GH3-1* and *GH3-4*) were found and duplication events of these six gene pairs were estimated to occur between 23.20 and 31.01 Mya. These results suggest that the expansion of the *GH3* gene family might be attributed to duplication events and this expansion could occur in the common ancestors of *Oryza* species, resulting in similar structures and characteristics among the existing *Oryza* species. The calculated divergence time (23.20–31.01 Mya) of these duplication events is earlier than the differentiation time (~14 Mya) of *Oryza* species, while it is close to the origin time (~23.9 Mya) of the rice tribe (*Oryzeae*) [47,48,49,50]. Thus, it can be further deduced that the *GH3* gene family produced these duplication events in a common ancestor of *Oryzeae*. Interestingly, tandem duplication events (*GH3-9* and *GH3-10*) were only found in *O. sativa* ssp. *japonica* and *O. nivara*. This may be evidence to support the double domestication hypotheses of Chinese-cultivated rice subspecies [49,51,52,53] that *O. sativa* ssp. *japonica* originated from *O. rufipogon* and *O. nivara* and that *O. sativa* ssp. *indica* originated from *O. rufipogon* [47].

The expression results of different tissues were consistent with previous findings on other species that *GH3* genes displayed tissue-specific expression profiles [10,54]. For example, *OsGH3-5* showed a high expression level in anther and shoots, implying that *OsGH3-5* is involved in anther and shoot development. *OsGH3-3* displayed relatively high expression levels in seed-5 DAP, which suggests that *OsGH3-3* may be associated with seed development and growth. In addition, *OsGH3-2*, *OsGH3-4*, and *OsGH3-8* had a high expression level in post-emergence inflorescence, indicating that these three genes may work together on post-emergence inflorescence development. Previous studies have reported that several *GH3* genes play crucial roles in biotic and abiotic stress response [5,6,7]. For example, the *SbGH3* gene was expressed at a low level under a normal condition, whereas it was substantially enhanced under salt and drought stress [55]. In chickpea *CaGH3-1* and *-7*, and in *Medicago MtGH3-7*, *-8*, and *-9*, expression levels were significantly enhanced under drought or/and salinity stress [7]. In this study, we also found that the expression levels of *OsGH3* genes were different under salinity stress. The majority of *OsGH3* genes showed upregulation at different time points after NaCl treatment, indicating that *OsGH3* genes play important roles in salinity stress response. However, some *OsGH3* genes showed different expression profiles in leaves and roots. It can be inferred that the *OsGH3* genes have different roles in roots and leaves. In addition, several *OsGH3* genes formed a co-expression relationship network. Coincidentally, several genes showed a similar expression trend in the same tissues after NaCl treatment, such as *OsGH3-5* and *OsGH3-6*, in leaves. These results indicated that *OsGH3* genes may collaborate with each other in the response to salinity stress. Importantly, *OsGH3-2* and *OsGH3-8* were significantly upregulated at all the tested points in leaves and roots. *OsGH3-1* was upregulated at all the tested points in leaves, while it was upregulated at only one time point in roots. Conversely, *OsGH3-9* and *OsGH3-12* were upregulated at all the tested points in roots, while they were upregulated at two time points in leaves. Based on these results, we speculate that *OsGH3-2* and *OsGH3-8* play important roles in leaves and roots and that *OsGH3-1* plays a greater role in leaves than in roots under salinity stress, while *OsGH3-9* and *OsGH3-12* play a greater role in roots than in leaves under salinity stress.

In summary, a systematic analysis of *GH3* in six *Oryza* species/subspecies was performed. The results revealed that the gene structure, conserved motifs, phylogenetic analysis, chromosome location, gene duplication events, and Ka/Ks ratios of the *GH3* family were strictly conservative among *Oryza* species. The expansion of the *GH3* family might be attributed to segmental duplication and tandem duplication, and this expansion could have occurred in the common ancestors of *Oryza* species and can be traced back to the origin time (~23.9 Mya) of the rice tribe (*Oryzeae*) [47]. The tandem duplication events (*GH3-9* and *GH3-10*) were only found in *O. sativa* ssp. *japonica* and *O. nivara*. This may be evidence to support the double domestication hypotheses of Chinese-cultivated rice subspecies [49]. Similar to previous reports [10,54], *OsGH3* genes showed tissue-specific expression. In addition, the qRT-PCR result indicated that *OsGH3* genes play vital roles under salinity stress.

## Figures and Tables

**Table 1:** *GH3* genes identified in six *Oryza* species/subspecies.

Species	Name	Gene Identifier	CHR	CHR.start	CHR.end	Subfamily
*Oryza sativa* ssp. *japonica*	OsGH3-3	LOC_Os01g12160.1	Oschr1	6,624,963	6,630,462	Group1
	OsGH3-5	LOC_Os05g50890.1	Oschr5	29,200,198	29,205,717	Group1
	OsGH3-6	LOC_Os05g05180.2	Oschr5	2,522,903	2,525,214	Group1
	OsGH3-12	LOC_Os11g08340.1	Oschr11	4,401,492	4,403,522	Group1
	OsGH3-1	LOC_Os01g57610.1	Oschr1	33,308,448	33,311,391	Group2
	OsGH3-2	LOC_Os01g55940.1	Oschr1	32,221,376	32,225,123	Group2
	OsGH3-4	LOC_Os05g42150.1	Oschr5	24,643,516	24,646,086	Group2
	OsGH3-7	LOC_Os06g30440.1	Oschr6	17,586,899	17,590,682	Group2
	OsGH3-8	LOC_Os07g40290.1	Oschr7	24,149,649	24,152,079	Group2
	OsGH3-9	LOC_Os07g38890.1	Oschr7	23,325,197	23,327,227	Group2
	OsGH3-10	LOC_Os07g38860.1	Oschr7	23,314,482	23,319,154	Group2
	OsGH3-11	LOC_Os07g47490.1	Oschr7	28,391,725	28,400,307	Group2
	OsGH3-13	LOC_Os11g32520.1	Oschr11	19,188,565	19,190,125	Group2
*Oryza sativa* ssp. *indica*	OiGH3-3	BGIOSGA002109-PA	Oichr1	7,084,891	7,089,830	Group1
	OiGH3-5	BGIOSGA020457-PA	Oichr5	30,569,921	30,572,557	Group1
	OiGH3-6	BGIOSGA018825-PA	Oichr5	2,768,250	2,770,394	Group1
	OiGH3-12	BGIOSGA034955-PA	Oichr11	4,242,522	4,244,552	Group1
	OiGH3-1	BGIOSGA004585-PA	Oichr1	36,754,839	36,756,946	Group2
	OiGH3-2	BGIOSGA004510-PA	Oichr1	35,535,345	35,538,565	Group2
	OiGH3-4	BGIOSGA017778-PA	Oichr5	26,098,760	26,100,739	Group2
	OiGH3-7	BGIOSGA021194-PA	Oichr6	18,602,739	18,606,054	Group2
	OiGH3-8	BGIOSGA023979-PA	Oichr7	22,260,570	22,262,481	Group2
	OiGH3-9	BGIOSGA024029-PA	Oichr7	21,410,892	21,414,347	Group2
	OiGH3-10	BGIOSGA025998-PA	Oichr7	21,402,461	21,402,912	Group2
	OiGH3-11	BGIOSGA023736-PA	Oichr7	26,392,482	26,397,014	Group2
	OiGH3-13	BGIOSGA03388PA	Oichr11	15,962,369	15,968,186	Group2
*Oryza rufipogon*	OrGH3-3	ORUFI01G08270.1	Orchr1	6,148,966	6,153,847	Group1
	OrGH3-5	ORUFI05G29440.1	Orchr5	25,858,361	25,864,126	Group1
	OrGH3-6	ORUFI05G03070.1	Orchr5	2,229,725	2,232,983	Group1
	OrGH3-12	ORUFI11G05180.1	Orchr11	4,022,556	4,024,586	Group1
	OrGH3-1	ORUFI01G36550.1	Orchr1	30,644,226	30,646,778	Group2
	OrGH3-2	ORUFI01G35260.1	Orchr1	29,632,340	29,636,282	Group2
	OrGH3-4	ORUFI05G23110.1	Orchr5	21,592,626	21,594,605	Group2
	OrGH3-7	ORUFI06G16960.1	Orchr6	16,046,446	16,049,761	Group2
	OrGH3-8	ORUFI07G21680.1	Orchr7	21,004,765	21,006,676	Group2
	OrGH3-9	ORUFI07G20520.1	Orchr7	20,243,069	20,244,953	Group2
	OrGH3-10	ORUFI07G20500.1	Orchr7	20,233,907	20,238,965	Group2
	OrGH3-11	ORUFI07G26860.1	Orchr7	24,893,286	24,900,088	Group2
	OrGH3-13	ORUFI11G16590.1	Orchr11	19,413,084	19,420,302	Group2
*Oryza nivara*	OnGH3-3	ONIVA01G09800.1	Onchr1	7,585,019	7,589,913	Group1
	OnGH3-5	ONIVA05G29530.1	Onchr5	27,396,671	27,402,904	Group1
	OnGH3-6	ONIVA05G02860.1	Onchr5	2,172,465	2,175,724	Group1
	OnGH3-12	ONIVA11G05730.1	Onchr11	4,716,885	4,718,915	Group1
	OnGH3-1	ONIVA01G38150.1	Onchr1	32,674,099	32,677,412	Group2
	OnGH3-2	ONIVA01G36390.1	Onchr1	31,322,609	31,326,729	Group2
	OnGH3-4	ONIVA05G22520.1	Onchr5	22,188,295	22,190,274	Group2
	OnGH3-7	ONIVA06G18950.1	Onchr6	17,364,605	17,367,911	Group2
	OnGH3-8	ONIVA07G19200.1	Onchr7	18,453,792	18,455,703	Group2
	OnGH3-9	ONIVA07G18070.1	Onchr7	17,588,411	17,591,865	Group2
	OnGH3-10	ONIVA07G18060.1	Onchr7	17,579,620	17,585,886	Group2
	OnGH3-11	ONIVA07G25530.1	Onchr7	23,284,878	23,289,419	Group2
	OnGH3-13	ONIVA11G14940.1	Onchr11	16,487,974	16,493,787	Group2
*Oryza punctata*	OpGH3-3	OPUNC01G07310.1	Opchr1	6,025,916	6,031,401	Group1
	OpGH3-5	OPUNC05G25060.1	Opchr5	30,476,518	30,480,678	Group1
	OpGH3-6	OPUNC05G02820.1	Opchr5	2,336,689	2,340,237	Group1
	OpGH3-12	OPUNC11G05160.1	Opchr11	4,742,463	4,744,418	Group1
	OpGH3-1	OPUNC01G32450.1	Opchr1	35,758,425	35,761,223	Group2
	OpGH3-2	OPUNC01G31080.1	Opchr1	34,433,992	34,437,877	Group2
	OpGH3-4	OPUNC05G19440.1	Opchr5	26,021,393	26,023,350	Group2
	OpGH3-7	OPUNC06G12520.1	Opchr6	13,614,325	13,618,401	Group2
	OpGH3-8	OPUNC07G19610.1	Opchr7	26,181,745	26,183,673	Group2
	OpGH3-9	OPUNC07G18650.1	Opchr7	25,197,946	25,199,823	Group2
	OpGH3-11	OPUNC07G24330.1	Opchr7	29,891,760	29,898,273	Group2
	OpGH3-13	OPUNC11G12860.1	Opchr11	20,394,638	20,400,442	Group2
*Oryza glumaepatula*	OgGH3-3	OGLUM01G08700.1	Ogchr1	7,724,249	7,730,000	Group1
	OgGH3-5	OGLUM05G28990.1	Ogchr5	29,839,316	29,844,943	Group1
	OgGH3-6	OGLUM05G02960.1	Ogchr5	2,520,030	2,523,301	Group1
	OgGH3-12	OGLUM11G05200.1	Ogchr11	4,202,889	4,204,936	Group1
	OgGH3-1	OGLUM01G37610.1	Ogchr1	36,364,851	36,367,411	Group2
	OgGH3-2	OGLUM01G36180.1	Ogchr1	35,099,335	35,107,582	Group2
	OgGH3-4	OGLUM05G23040.1	Ogchr5	25,041,853	25,043,832	Group2
	OgGH3-8	OGLUM07G20630.1	Ogchr7	23,059,911	23,061,822	Group2
	OgGH3-9	OGLUM07G19490.1	Ogchr7	22,131,580	22,133,468	Group2
	OgGH3-10	OGLUM07G19470.1	Ogchr7	22,122,128	22,126,771	Group2
	OgGH3-11	OGLUM07G25930.1	Ogchr7	27,048,553	27,056,767	Group2
	OgGH3-13	OGLUM11G14980.1	Ogchr11	18,442,620	18,448,411	Group2

Note: CHR/chr in Table 1 represents chromosome. Os represents *Oryza sativa* ssp. *japonica*. Oi represents *Oryza sativa* ssp. *indica*. Or represents *Oryza rufipogon*. On represents *Oryza nivara*. Op represents *Oryza punctata*. Og represents *Oryza glumaepatula*. The full length of BGIOSGA025998-PA is obtained by integrating BGIOSGA025998-PA and BGIOSGA025998-PA based on the R498 genome annotation.

**Table 2:** Ka, Ks, and Ka/Ks values for the duplication gene pairs from six *Oryza* species/subspecies.

Seq1	Seq2	Ks	Ka	Ka/Ks Ratio	Date (MY)	Duplication Type
OiGH3-1	OiGH3-4	0.5644	0.207	0.367	31.010989	WGD or segmental duplication
OsGH3-1	OsGH3-4	0.4274	0.1251	0.293	23.483516	WGD or segmental duplication
OgGH3-1	OgGH3-4	0.4223	0.1273	0.301	23.203297	WGD or segmental duplication
OnGH3-1	OnGH3-4	0.4237	0.1269	0.300	23.28022	WGD or segmental duplication
OpGH3-1	OpGH3-4	0.4754	0.1245	0.262	26.120879	WGD or segmental duplication
OrGH3-1	OrGH3-4	0.4198	0.1252	0.298	23.065934	WGD or segmental duplication
OiGH3-10	OiGH3-9	0.2407	0.1365	0.567	13.225275	Tandem duplication
OnGH3-10	OnGH3-9	0.25	0.1274	0.510	13.736264	Tandem duplication

## Supplementary Materials

The following are available online at http://www.mdpi.com/2223-7747/8/2/30/s1, Table S1: Primers of *OsGH3* genes for qRT-PCR in this study. Table S2: The homologous analyses of *GH3* genes among different species were carried out using MCscanX software. Table S3: The FPKM (fragments per kilo base of exon per million fragments mapped) values of *OsGH3* genes in different tissues.
